# Lesbian and Heterosexual Women’s Implicit Responses to Gender Figures

**DOI:** 10.1007/s10508-024-02972-9

**Published:** 2024-09-03

**Authors:** José Cláudio Rodrigues da Silva, Rodrigo Vianna de Almeida, Renato Bortoloti

**Affiliations:** 1 Department of Psychology, Universidade Federal de Minas Gerais, Belo Horizonte, Brazil; 2 School of Psychology, Ulster University, Cromore Road, Coleraine, BT52 1SA Northern Ireland; 3 Instituto Nacional de Ciência e Tecnologia Sobre Comportamento, Cognição e Ensino, São Paulo, Brazil

**Keywords:** Sexual orientation, Implicit Relational Assessment Procedure, Functional Acquisition Speed Test, Female sexuality, Lesbian, Implicit measures

## Abstract

This study explored how heterosexual and lesbian women differ in their implicit sexual responses. Previous research indicates that heterosexual women have physiological and implicit responses to both genders, whereas lesbian women show stronger responses to their preferred gender. This study used two implicit measures: the Implicit Relational Assessment Procedure (IRAP) and the Function Acquisition Speed Test (FAST), both of which were novel in this context. We recruited 33 heterosexual and 25 lesbian women. Both IRAP and FAST were successful in differentiating the two sexual orientations as a group. The results confirmed that heterosexual women exhibit positive responses to both genders, while lesbian women show stronger, category-specific responses to their preferred gender. These findings align with previous research and provide further insight into the nuanced differences in sexual orientation responses among women.

## Introduction

Sexual orientation and sexual responses may not always perfectly align (Coleman, 1987; Colledani & Ciani, 2021; Snowden et al., 2024; Zivony, 2020). For example, heterosexual women do not show category-specific genital responses to sexual stimuli, usually presenting similar genital arousal to both male and female erotic stimuli (Chivers, 2017; Chivers et al., 2015). In contrast, these responses are category-specific in lesbians (Chivers et al., 2004, 2007, 2010). Category-specific arousal is also observed in both gay and heterosexual men (Chivers et al., 2004; Freund, 1963; Snowden et al., 2008). These studies suggest that heterosexual women are, on average, physiologically sexually aroused to both male and female sexual stimuli, whereas lesbians tend to be more aroused to their preferred sex, a pattern typically seen in men. However, the male-typical sexual arousal in lesbians is independent of masculine behaviors, suggesting the existence of several gendered dimensions within sex (Rieger et al., 2016). Apart from physiological responses, several implicit methods have been employed in the context of sexual attraction to investigate early appraisal of sexual stimuli, including viewing times (Imhoff et al., 2010), Stroop interference (Ó Ciardha & Gormley, 2013), choice reaction times (Wright & Adams, 1994), rapid serial visual presentation (Zappala et al., 2013), gaze patterns (Dawson et al., 2017), dot-probe tasks (Snowden et al., 2016), the Implicit Association Test (Snowden & Gray, 2013), and the Implicit Relational Assessment Procedure (IRAP) (Rönspies et al., 2015; Timmins et al., 2016). Advantages of such implicit measures include their non-invasiveness (see Babchishin et al., 2013; Snowden et al., 2008; Timmins et al., 2016), their potential to capture subtle effects (see Chivers et al., 2010; Dawson et al., 2009; Hinzmann et al., 2020), and the possibility of directly comparing sexes despite different anatomical configurations (see Holmes et al., 2021; Rieger & Savin-Williams, 2012). The IRAP, for example, has the advantage of enabling to observe responses to specific combinations of stimuli differentially.

The IRAP (Barnes-Holmes et al., 2008) requires participants to alternate between responding in two ways: one that coheres with their previous learning history and one that contradicts it. Each corresponds to a block of trials. For instance, in some blocks, participants might be asked to relate flowers to positive attributes, while in other blocks, they might be asked to relate flowers to negative attributes. The IRAP effect is identified by shorter response latencies in blocks of trials where participants respond in a manner that is coherent with their learning history, as compared to longer response times in blocks of trials that contradict their learning history. Typically, each IRAP trial presents a label (e.g., a flower), a target (e.g., a word with positive connotations), and two response options (e.g., “Similar”/“Different”, “True”/“False”, or “Yes”/“No”). In an IRAP focusing on reactions to flowers, for example, a trial might present “daisy” as the label, “affection” as the target, and “True”/“False” as response options. In one block of trials, responding “True” to “daisy” and to “affection” is reinforced (i.e., this would be the correct response), whereas in the other block of trials, “False” would be reinforced as the correct response. The former is deemed coherent with participants’ likely learning history, and the second is deemed incoherent with the most likely learning history. Based on the differences in reaction times between history-coherent and history-incoherent blocks (calculated as effect sizes called *D*_IRAP_ scores), the IRAP has been shown to differentiate groups across a variety of domains, including social identities (Hughes et al., 2017), vegetarian status (Barnes-Holmes et al., 2010b), gender stereotypes in children (Rabelo et al., 2014), and, in fact, men’s sexual orientation (Rönspies et al., 2015; Timmins et al., 2016).

The IRAP was employed by Timmins et al. (2016) to investigate category-specific attraction in gay and heterosexual men. Researchers operationally defined participants as gay or heterosexual men based on their stable patterns of same or opposite gender sexual partners, respectively. A modified version of the Klein Sexual Orientation Grid (KSOG) was employed to assess those patterns (Klein, 1993; Klein et al., 1985; Weinrich et al., 1993). The KSOG results confirmed that all participants predominantly fell into one of these two categories. In the IRAP trials, participants were presented with a male or female nude image at the top of the screen, accompanied by either a positive (e.g., “arousing”) or negative (e.g., “repulsive”) target word in the middle. The bottom of the screen presented the response options “True” and “False” on the bottom left and right corners of the screen. The side of the screen on which the response options would appear was randomly reassigned from trial to trial. This setup led to four types of trials in Timmins et al.’s IRAP: Female–Attractive, Female–Unattractive, Male–Attractive, and Male–Unattractive. Each block involved a predetermined rule participants were required to respond according to. Blocks had thus either a “Straight-like” rule (i.e., respond as if all females are attractive and not unattractive, and all males are unattractive and not attractive) or a “Gay-like” rule (i.e., respond as if all males are attractive and not unattractive, and all females are unattractive and not attractive). A “Gay-like” block always followed a “Straight-like” block and vice versa. The starting block was randomly counterbalanced across participants.

The patterns of responses in the IRAP revealed that heterosexual men displayed a marked preference for female images, contrasted with an aversion to male images. Conversely, gay men exhibited a pronounced preference for male images (Timmins et al., 2016). This is inferred from the IRAP effects; that is, differences in reaction times to respond to one type of block versus the other. IRAP effects have been shown to correspond to explicit preferences (Power et al., 2009), thereby rendering them implicit bias estimators (cf. Barnes-Holmes & Harte, 2022; see also Cullen & Barnes-Holmes, 2008). Using the area under the receiver operating characteristic (ROC) curve, Timmins et al. showed that the overall IRAP effect, as well as the IRAP effects for erotic images of both males and females, were significantly successful in identifying heterosexual and gay participants. Their sexuality, as measured by the KSOG and explicit self-reports, was significantly and strongly correlated with these three IRAP effects. Hence, both groups of participants produced IRAP scores that aligned with their sexual orientation. Nevertheless, gay men did not display an IRAP effect for pictures of women. Timmins et al. interpreted this finding in light of the fact that the IRAP is sensitive to attitude learning (Hughes & Barnes-Holmes, 2011) and the media exposes females as sexual objects (Lin, 1998).

**Fig. 1:**
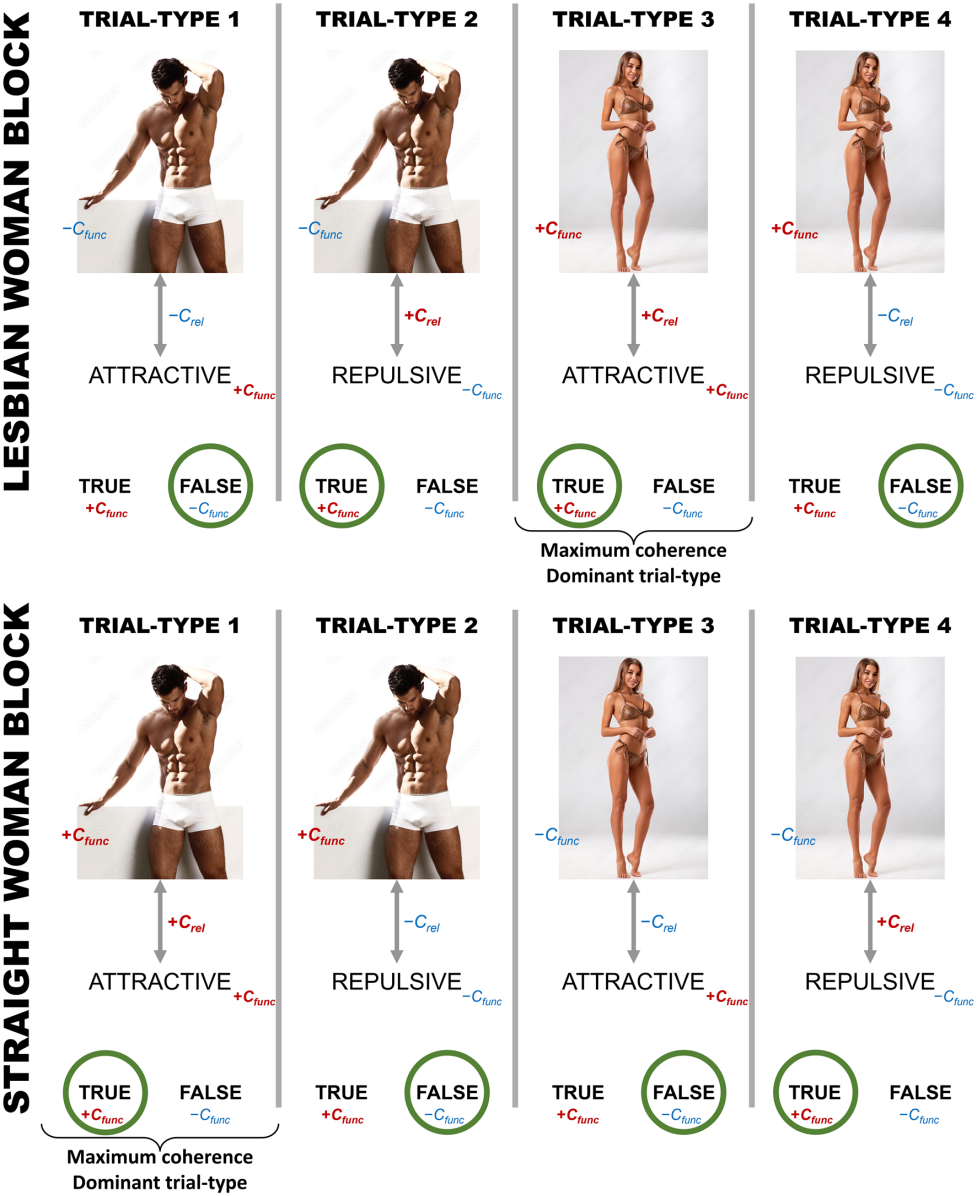
The DAARRE model exemplifying the IRAP configuration employed in the present study. *Note*. The DAARRE model employs positive (+) and negative (−) qualifiers to study the relative role of each *C*_rel_ and *C*_func_ within the stimuli network presented in an IRAP (Harte & Barnes-Holmes, 2024). Trial-types were presented randomly within each block, for 6 times each (randomly combining one out of the three label stimuli with one of the three target stimuli), totaling 24 trials per block. Both blocks were presented to all participants in a sequence such that one followed the other (the starting block that determined the following order was randomly assigned). In the present study, the “Lesbian woman block” was coherent with the history and orientation of people who love women, and the “Straight women block” was coherent with the history and orientation of people who love men. The two model pictures were extracted from Adobe Stock: photo #70616915 is “Sexy Portrait Male Model in Underwear” by ASjack, and photo #313314673 is “Sexy Girl in Bikini Isolated on the Gray Background” by nikolas_jkd. These pictures were not used in the IRAP

The study by Timmins et al. (2016) was conducted before the formal introduction of a recent analytical framework for the IRAP results, known as the differential arbitrarily applicable relational responding effects (DAARRE) model (Finn et al., 2018). This model highlights the functional characteristics of stimuli as applied to the IRAP, in addition to the relational characteristics between stimuli previously emphasized in interpreting IRAP results (Barnes-Holmes et al., 2010a). These functional characteristics encompass stimuli attentional, emotional, and motivational impacts upon relational responding. It suggests that individuals engaging in an IRAP might be attuned not only to the semantic connection between label and target stimuli, but also to the shared functional attributes of stimuli and response options, thereby providing relative higher or lower coherence per trial-type. Acknowledging the influence of both functional and relational properties of stimuli has enhanced the understanding of IRAP effects in numerous recent investigations (e.g., Bortoloti et al., 2023; de Almeida et al., 2024; Finn et al., 2019; Kavanagh et al., 2018, 2019; see Fig. 1 for an illustration of how the DAARRE model apply to the IRAP configuration employed in the present study).

According to the DAARRE model, the results observed by Timmins et al. (2016) could be interpreted in light of the nude female images having greater orienting and evoking appeal for heterosexual men, while nude male images held similar appeal for gay men. Given that this orienting and evoking appeal is shared with the positive erotic words, the DAARRE model would describe the trial types featuring nude females as highly coherent with erotic words in the history of heterosexual men. Similarly, nude males would be highly coherent with erotic words in the history of gay men (see Barnes-Holmes et al., 2020). In essence, the relational (*C*_*rel*_) and functional (*C*_*func*_) properties of stimuli—including the word representing the correct response option—cohered when heterosexual men needed to respond “True” to female pictures with positive adjectives and when gay men needed to respond “True” to male pictures and positive adjectives, thereby generating a distinct dominant trial-type for each group. Incoherence arose when heterosexual men needed to relate as “True” male images with erotically evoking adjectives, but the same incoherence did not necessarily happen when gay men needed to relate as “True” female images with erotically evoking adjectives.

Drawing on this speculative DAARRE model explanation, the IRAP trial-types involving female images were thus sensitive to both sexual orientation and an attitudinal learning history (Hughes & Barnes-Holmes, 2011). This interpretation arose from the report by Timmins et al. (2016) which presented the two trial-types involving male images combined (Male–Attractive and Male–Unattractive), as well as the two trial-types involving female images combined (Female–Attractive and Female–Unattractive). The IRAP also differentiated heterosexual and gay men in the study by Rönspies et al. (2015), in which IRAP effects for all four trial-types were reported separately. Rönspies et al. used the Kinsey et al. (1948) scale to identify participants as gay or heterosexual men, and used the same trial-type structure of Timmins et al., along with other measures (viewing time and choice reaction time) to assess the IRAP’s validity in capturing sexual orientation. Using the area under the ROC curve and correlations, Rönspies et al. showed the IRAP to be tightly convergent with viewing time (VT) and the Kinsey scale. The authors concluded for “substantial evidence for the criterion validity of the VT and IRAP” (p. 1489). Interestingly, in line with Timmins et al., Rönspies et al. observed no particular bias for relating women with sexually unattractive attributes from gay men’s response patterns. This is one level more specific than the report by Timmins et al., in which gay men’s responses to female pictures were collapsed across trials with positive and negative attributes. It seems that gay men responded equally when requested to confirm and disconfirm aversive attributes to females, but disconfirmed more promptly than confirmed sensual attributes to females.

Rönspies et al. (2015) also observed that heterosexual men confirmed sensually positive attributes to males more promptly than disconfirmed (less so than did gay men, but still significantly). The authors interpreted that, in heterosexuality, both males and females are involved in the sexual pursuit, and thus men’s reckoning of the sensuality of fellow heterosexual men may be more relevant to them than, for that matter, gay men’s reckoning of the sensuality of females (or, speculatively, the same could happen for gay men’s appraisal of women as competitors for men, as results may indicate an attentional effect to female sensuality by gay men). This finding in heterosexual men is aligned with the category-non-specific characteristic of heterosexual women, which highlights the importance of conducting similar research with heterosexual and lesbian women. Because attitudinal learning seems important in “interfering” with response patterns based on sexual orientation, it seems relevant to include estimates of behavior acquisition rates. To that end, an instrument that implicitly measures the impact of the previous learning history on novel learning rates is the Function Acquisition Speed Test (FAST) (Cartwright et al., 2016; Cummins et al., 2018).

The FAST is comprised of two blocks of trials presented in a random order. One block, the “consistent” block, aligns with the participant’s learning history, while the other, the ‘inconsistent’ block, does not. Each trial features a single stimulus at the center of a blank screen with two possible keyboard response options. These stimuli can be said to belong to one of four types: two types of attribute stimuli (e.g., positive and negative adjectives, like “arousing” and “repulsive”), and two types of category stimuli (e.g., male and female nude pictures). Participants are briefed to learn to respond to these stimuli using one of two designated keys on their keyboard (e.g., “left” and “right”). Correct responses receive positive feedback, while incorrect or missed ones receive negative feedback. The instructions clarify that participants should learn the correct responses based on post-response feedback. In the consistent block, stimuli from compatible categories require the same response key, whereas those from incompatible categories require different keys. The inconsistent block reverses this: stimuli from compatible categories require different keys, while those from incompatible categories use the same key. The FAST score is derived by calculating the difference between the learning curves’ slopes for the consistent and inconsistent blocks. This score reflects the participant’s adaptability to the response demands between the two blocks. Therefore, in effectively teaching new, arbitrary responses to one stimulus at a time, the FAST uses learning curve slopes as a proxy for the ease or difficulty to acquire new behavioral functions. It is thus possible to identify the stimuli for which participants found it easier to share the same arbitrary response, thereby implying that these stimuli had been more coherently related in participants’ previous learning history.

By comparing learning acquisition ease (as measured by the FAST) and differences in stable patterns of relational responding (as measured by the IRAP), the present study aims to respond to the speculations of Rönspies et al. (2015) whereby, in involving two genders, heterosexuality may be more sensitive to the sensuality of same-gender peers, and of Timmins et al. (2016), whereby exposure to social attitudes to women may influence responses to nude females. Both these studies were limited in only including men, thereby leaving the question as to whether these interpretations would hold similarly for women, considering, as mentioned, that heterosexual women often do not show gender-specific genital responses (Chivers, 2017; Chivers et al., 2007, 2010).

The present study explores the use of the IRAP and the FAST (measures of distinct processes but both sensitive to previous learning histories) in disentangling the sexual biases of heterosexual and lesbian women. Our approach is underpinned by the nuanced insights of the DAARRE model, which integrates the overall functional characteristics of stimuli, encompassing their attentional and emotional impacts on relational responding. As of yet, exploring these conjectures in a female cohort remains largely uncharted territory. Applying both implicit measures to female participants in light of the previous results with male participants could offer new insights on the interplay between category-specific attraction and attitudinal learning exposure across sexualities and genders.

## Method

### Participants

Participants were 58 undergraduate students enrolled in a Brazilian university. All of them were females aged between 18 and 25 years. They were grouped according to a self-declared stable pattern of sexual partners. Group 1 was comprised of 33 heterosexual women (operationally defined as “women with relatively stable preference for sexual partners of the opposite sex”—Snowden et al., 2008). Group 2 was comprised of 25 lesbian women (operationally defined as “women with relatively stable preference for same-sex sexual partners”—Snowden et al., 2008). This “stable preference” was confirmed by a modified version of the Klein Sexual Orientation Grid—KSOG (Klein, 1993; Klein et al., 1985). According to the inclusion criterion, all participants had to be either heterosexual or lesbian females: therefore, recruited volunteers who declared to be bisexual or asexual were thanked and debriefed. All participants had typical or corrected vision and two functional hands. They were previously informed about the research objectives and methods and signed an informed consent form in order to participate.

### Procedure

Data collection was performed individually, in a room specially reserved for this activity, with relative isolation from external interferences. Computerized procedures (IRAP and FAST) were run on an HP laptop with a 15’’ screen (1024 × 768 pixel resolution). The application order of the procedures was the same for all participants: first the KSOG, second the IRAP, and third the FAST.

### Measures

#### The Klein Sexual Orientation Grid (KSOG)

The KSOG presents seven sexual orientation components on an answer sheet. Three columns indicate the three different reference points on which sexual orientation is addressed: the participant’s past, her present, and her ideal. Using a 7-point scale, the participant rated (from 1, other sex only, to 7, same sex only) each of the 21 combinations between the seven components (i.e., items) and the three reference points. Averaging out the 7-point scale scores across the three columns results in a classification of sexual orientation whereby a score of 1 corresponds to a strictly heterosexual individual, 3.5 to a strictly bisexual individual, and 7 to a strictly homosexual individual. Participants responded to a translation to Portuguese of the original KSOG in English.

#### The Implicit Relational Assessment Procedure (IRAP)

The IRAP was prepared following the overall description by Barnes-Holmes et al. (2010a). Before starting the procedure, the participants were instructed to respond as accurately and quickly as possible. They were also instructed to carefully read the instructions presented before each block, indicating the expected response pattern for that block, which could be consistent or inconsistent with their learning history.

Three nude male photographs (IDs: 4500, 4538, 4561) and three nude female photographs (IDs: 4141, 4142, 4225) selected from the International Affective Picture System (IAPS) were used as label stimuli. These images had comparable levels of valence (4500: *M* = 5.70, *SD* = 2.12; 4538: *M* = 5.91, *SD* = 2.03; 4561: *M* = 5.02, *SD* = 2.28; 4141: *M* = 5.59, *SD* = 2.46; 4142: *M* = 5.45, *SD* = 2.82; 4225: *M* = 6.09, *SD* = 1.82) and arousal (4500: *M* = 3.68, *SD* = 2.42; 4538: *M* = 4.65, *SD* = 2.63; 4561: *M* = 4.35, *SD* = 2.67; 4141: *M* = 5.25, *SD* = 2.48; 4142: *M* = 5.60, *SD* = 2.61; 4225: *M* = 5.39, *SD* = 2.38), based on the IAPS Affective Ratings of Pictures (Lang et al., 2008). In addition, the Portuguese equivalent to the words attractive, sensual, exciting (positive terms) and uninteresting, turn off, repulsive (negative terms), serving as target stimuli, were presented along with the pictures in IRAP trials. In each trial, a picture (label), a word (target), and two response options—“V” (for True^1^) and “F” (for False)—were presented on the computer screen. Pictures of nude males or females were shown at the top of the screen, positive or negative terms were presented at the center, and the response options “V” (true) and “F” (false) were presented at the bottom of the screen on either side. The four possible combinations between two types of label stimuli (male or female pictures) and two types of target stimuli (positive or negative words) were randomly presented for six times each within a block of 24 trials, thereby generating four trial-types (Fig. 1): trial-type 1 (male–attractive); trial-type 2 (male–unattractive); trial-type 3 (female–attractive); trial-type 4 (female–unattractive).

In a “Straight woman” block of trials, the participant was instructed to select the option “True” when a male picture was presented with a positive term and the option “False” when a male picture was presented with a negative term. She was also instructed to choose “False” for the combination of a female picture with a positive term and “True” for a female picture with a negative term. Therefore, in the “Straight woman” blocks, the required responses were: trial-type 1: “True,” trial-type 2: “False,” trial-type 3: “False,” and trial-type 4: “True.” In a “Lesbian woman” block of trials, the participant was instructed to respond in the opposite way to that required in the “Straight woman” blocks. Therefore, in the “Lesbian woman” blocks, the required responses were: trial-type 1: “False,” trial-type 2: “True,” trial-type 3: “True,” trial-type 4: “False.” See Fig. 1 for a schematic presentation of the four IRAP trial-types in light of the DAARRE model.

All blocks were presented in pairs. A pair of blocks corresponds to one “Straight woman” block and one “Lesbian woman” block, thereby generating an alternate sequence of blocks wherein a “Straight woman” block followed a “Lesbian woman” block or vice versa. The starting block determined the following order of the sequence, which was randomized across participants (i.e., half started with the “Straight woman” block, and half started with the “Lesbian woman” block). All blocks encompassed 24 trials, with each trial-type presented randomly for six times per block. The IRAP started with one pair of practice (training) blocks that required the participant to meet the performance criteria. Participants were expected to achieve at least 80% of correct responses within a median response time of less than 2000 ms (ms) in each block. If the participant did not meet these criteria, she would be re-exposed to the practice blocks until she did. Participants had up to three pairs of training blocks to achieve the performance criteria.

For each trial, choosing the option considered correct removed the stimuli presented on the screen in that trial—then, after a 400-ms intertrial, the next trial started. If the participant chose the option considered incorrect for the current block, a red “X” was presented at the center of the screen, and the participant did not move on to the next trial until she pressed the correct response key. Feedback on accuracy and median response time was presented on the screen at the end of each block. Once the participant met the performance criteria in pairs of practice blocks, she proceeded to respond to exactly three pairs of test blocks. The procedure for the test blocks was similar to the practice blocks; however, no performance criteria were required from the participant to move on to the next pair of blocks (i.e., all successful participants undertook exactly three pairs of test blocks regardless of their accuracy and latency, but they were still under feedback pressure). Participants were not explicitly told that the performance criteria was no longer required to finish the sequence of blocks. Only the data recorded in the test blocks were considered in the analyses presented in this study. Participants who did not reach the minimum performance criteria had no *D*_IRAP_ score data (i.e., they had missing IRAP data), but had data in the other measures (KSOG and FAST).

In the following, the IRAP procedure is summarized. At each trial (i.e., a screen to which participants had to provide a response in order to move to the next trial), participants were presented with a random combination of label (top-screen stimulus) and target (mid-screen stimulus), and always the same two response options (bottom screen stimuli, on both sides). One out of the three exemplars of male pictures were shown as label in trial-types 1 and 2, and one out of the three exemplars of female pictures were shown as label in trial-types 3 and 4 (selection of one out of the three pictures was random). One out of the three exemplars of positive words were shown as target in trial-types 1 and 3, and one out of the three exemplars of negative words were shown as target in trial-types 2 and 4 (random selection of word out of the three). Each trial-type was repeated for six times per block. The order of presentation of trial-types within a 24-trial block was random. There are no concerns regarding order effects because participants had to respond quickly to these 24 trials per block across six test-blocks (three “Straight woman” blocks and three “Lesbian woman” blocks in an alternate sequence), after the presentation of at least one pair of practice blocks, and the dependent measure obtained from the IRAP (i.e., the *D*_IRAP_ scores) summarizes data from all trials. All sets of stimuli were presented for an equal number of types, thereby removing the possibility of unbalanced exposure to stimuli. Previous IRAP research has consolidated this configuration (Hughes & Barnes-Holmes, 2011; Hughes et al., 2011, 2012; Murphy et al., 2022; Vahey et al., 2015).

#### The Function Acquisition Speed Test (FAST)

The FAST was administered on the same computer used for the IRAP. The protocol followed the modifications adopted by Cartwright et al. (2016) and Cummins et al. (2018), consisting of one block of practice and two blocks of test. All blocks contained two distinct categories of stimulus. The test blocks employed the same pictures and words used in the IRAP. The test blocks were identical in format, differing only in terms of the reinforcement contingencies that were applied. Specifically, the “Straight woman” test block reinforced pressing a specific key for male pictures and for positive terms, and a different key for female pictures and for negative terms. The “Lesbian woman” test block rearranged these contingencies and reinforced pressing a key for male pictures and for negative terms, and another key for female pictures and positive terms. These two blocks were presented in random order across participants. Before starting the FAST blocks, the following instructions describing the general nature of the task were presented on the screen (in Portuguese):

In the section that follows, your task is to learn which button to press when a word or a picture is presented on the screen. IMPORTANT: during this phase you only have to press the “Z” key or the “M” key. Locate them on the keyboard now. To help you learn, you will receive feedback indicating whether you are right or wrong. If you have any question, ask the researcher now. Press any key when you’re ready to start.

After participants pressed a random key, they were presented with a practice block consisting of 16 trials, followed by two test blocks, each containing 50 trials. Identical instructions to those mentioned above were provided before each block. Reinforcement feedback (“CORRECT” or “WRONG”, in Portuguese) was presented in red on the screen for 1.5 s after a response was emitted. If no response was emitted within 3 s, a timeout response (“WRONG”) was shown at the center of the screen, also in red. Intertrial intervals took 250 ms. After completing all the FAST blocks, a written message indicated the end of the experiment. Participants were then thanked and debriefed.

### Data Analysis

The most important IRAP datum is the *response latency*, defined as the time in milliseconds (ms) that elapses between trial onset and the correct response is emitted by the participant. The IRAP latency data were converted into *D*_IRAP_ scores, which are calculated as effect sizes (i.e., the difference between average latencies in the two types of blocks is divided by the pooled standard deviation across blocks). This calculation intends to mitigate the impact of random individual factors such as age, motor skills, and cognitive ability (Greenwald et al., 2003).

All latency data were processed by the *D*_IRAP_ algorithm, available in the GO-IRAP software (https://balc-i.net/software-and-materials/). The algorithm processed the data as follows: (1) Latencies obtained from training blocks were discarded, and only latencies from tests blocks were used; (2) latencies above 10,000 ms were excluded from the analyses; (3) participants who presented more than 10% of test-block trials with latencies shorter than 300 ms had their data excluded from the study; (4) standard deviations for the four trial-types were computed: four across the response latencies from Test Blocks 1 and 2, four across Test Blocks 3 and 4, and four across Test Blocks 5 and 6, yielding a total of 12 standard deviations; (5) 24 mean latencies were calculated, one for each trial-type in each test block; (6) difference scores were calculated for each of the four trial-types separately, for each pair of test blocks, by subtracting the mean latency of the “Straight woman” block from the mean latency of the “Lesbian woman” block (this entails that positive scores will indicate shorter latencies in responding as a straight woman, and negative scores will indicate shorter latencies in responding as a lesbian woman); (7) each difference score was divided by its corresponding standard deviation calculated in step 4, thereby generating one *D*_IRAP_ score for each trial type, for each pair of test blocks, totaling 12 *D*_IRAP_ scores; (8) four *D*_IRAP_ scores, one for each trial-type, were calculated by averaging the scores for each trial type across the three pairs of test blocks (Timko et al., 2010).

By establishing the direction of the subtraction in the manner presented in step 6 (i.e., latency averages from the “Lesbian woman” block minus latency averages from the “Straight woman” block), positive *D*_IRAP_ scores indicate faster responding in the “Straight woman” blocks, whereas negative *D*_IRAP_ scores indicate that participants responded faster in the “Lesbian woman” blocks. The larger the *D*_IRAP_ score, the larger the difference in response latencies between these types of blocks (and/or the smaller the pooled standard deviation).

The main datum from the FAST was the learning rate, derived from the cumulative record of correct responses from participants. More specifically, a cumulative record was constructed for the “Lesbian woman” and “Straight woman” blocks separately by plotting the cumulative number of correct responses per block as a function of trial order. The learning rate corresponds to the slope of the regression line of best fit adjusted to the learning curve. Therefore, a larger slope indicates a more rapid learning rate. The learning rate differential (i.e., the difference in slope coefficients between the “Straight woman” and “Lesbian woman” blocks) was quantified by subtracting the slope of the “Lesbian woman” block from that of the “Straight woman” block. By calculating the FAST Slope Difference scores this way (i.e., “Straight woman” block slope minus “Lesbian woman” block slope), positive differences indicate a higher (i.e., “easier”) learning rate in combining males with positive adjectives and females with negative adjectives as compared to the learning rate in the opposite block, whereas negative differences indicate a higher learning rate in combining females with positive adjectives and males with negative adjectives as compared to the learning rate in the opposite block.

The data analysis script for the present study can be found at https://osf.io/kapq7. The data can be found at https://osf.io/4vybr/. All analyses were done in *R* (R Core Team, 2021). The script starts by testing some of the most common assumptions, namely: normality, homoscedasticity, autocorrelation, and multicollinearity. The functions used were, respectively, “Shapiro.test”, “LeveneTest”, “DurbinWatsonTest”, and “vif”, the latter three from the *car* package (Fox & Weisberg, 2019). It was observed that all assumptions were met, except for normality of heterosexual participants’ data in (1) *D*_IRAP_ scores at trial-type 4, (2) FAST Slopes at the “Straight woman” block, and (3) FAST Difference scores. Also, FAST slopes, per block and orientation, is heteroscedastic. The type of inferential tests (i.e., parametric or not) was then chosen according to whether or not the assumptions were met. When *D*_IRAP_ scores were averaged out across the four trial-types, they were normally distributed at either level of sexual orientation.

Across all analyses, the only factor in common was the between-subjects sexual orientation dichotomous factor, apart from the random-effects factor Participants. *D*_IRAP_ scores were analyzed as repeated measures when each trial-type was considered (within-subjects factor with four levels), but this factor was dropped when *D*_IRAP_ scores were averaged out across the four trial-types. After the presentation of descriptive statistics and data visualization plots, the first variable submitted to inferential tests was FAST Slope, followed by FAST slope differences. Because of failures in meeting assumptions, the nonparametric Wilcoxon rank sum exact test was employed to test for the difference in FAST Slope per block for each level of sexual orientation, and, for FAST slope differences (slopes already subtracted per block), between the two levels of sexual orientation (with our participants, given *α* = 0.05 and 1−*β* = 0.8, we could identify a Cohen’s *d* as small as 0.86, or Cliff’s *δ* ≈ 0.35). Then, the effect sizes were estimated using a version^2^ of the *r* statistic (*r* = Wilcoxon’s *W* ∕ (*n*_Straight_ × *n*_Lesbian_)) and Cliff’s *δ* based on the “cliff.delta” function from the *effsize* package (Torchiano, 2020).

The analyses proceed to a mixed-effects ANOVA where *D*_IRAP_ scores were the dependent variable, the four trial-types encompassed a repeated-measures within-subjects factor, sexual orientation was a between-subjects factor with two levels, and the participants themselves were a random-effects factor. The only assumption violation in *D*_IRAP_ scores was normality at the level of heterosexuals’ trial-type 4 *D*_IRAP_ scores, marginally (Shapiro–Wilk’s *p* = 0.10). We therefore proceeded with parametric analyses. We fitted a linear mixed-effects model by Restricted Maximum Likelihood using the “lmer” function from the *lme4* package (Bates et al., 2015). With support from the *lmerTest* package (Kuznetsova et al., 2017), we adopted the Satterthwaite’s method to estimate main effects and the interaction effect. Using the *emmeans* package (Lenth, 2024), confidence intervals for marginal means were estimated with Bonferroni adjustments, and pairwise comparisons were calculated using Tukey familywise correction in within-subjects comparisons, all with Kenward–Roger degrees-of-freedom estimations. Given a final sample of 48 participants (i.e., after 10 did not meet performance criteria in IRAP test blocks), for a mixed within-between ANOVA with four repeated measurements and two groups, given *α* = 0.05 and 1 − *β* = 0.8, we could estimate an effect size as small as Cohen’s *f* = 0.17, which is equivalent to Cohen’s *d* ≈ 0.24 or *η*^2^_p_ ≈ 0.028 (i.e., an effect size just over small).

In studying between-subjects differences in the FAST and the IRAP separately, we attempted to compare them to one another. Because the FAST does not allow for specific combinations of stimuli (whereas the IRAP trial-types do), we averaged out the *D*_IRAP_ scores across trial-types in order to compare the IRAP directly with the FAST. The averaged *D*_IRAP_ scores did follow a normal distribution, so we compared heterosexual and lesbian *D*_IRAP_ scores using a *t* test and Cohen’s *d* (given 80% power, we could identify *d* as small as 0.84). However, we also performed the same nonparametric tests that we submitted FAST slope differences to (i.e., Wilcoxon’s rank-sum *W* for difference and, for effect sizes, *r* and Cliff’s *δ*), in order to directly compare the averaged *D*_IRAP_ scores’ results with those of the FAST.

Using the *pROC* package (Robin et al., 2011), we obtained eight Receiver Operating Characteristic (ROC) curves, each between sexual orientation and one of the dependent variables, namely: FAST slope differences, averaged *D*_IRAP_ scores across the four trial-types, trial-type 1 *D*_IRAP_ scores, trial-type 2 *D*_IRAP_ scores, trial-type 3 *D*_IRAP_ scores, trial-type 4 *D*_IRAP_ scores, male picture trial-types *D*_IRAP_ scores (average between trial-types 1 and 2), and female picture trial-types *D*_IRAP_ scores (average between trial-types 3 and 4). In these curves, sensitivity (i.e., true positive rate), in the *y*-axis, was a function of specificity (i.e., true negative rate, rather than the false positive rate), which ranged from 1 to 0 along the *x*-axis. We employed DeLong’s test to compare the averaged-out *D*_IRAP_ score and the FAST slope differences ROC curves directly.

Finally, after checking for the assumptions for the KSOG (which is not normally distributed, as the distribution looks bimodal and is discontinued between lesbian and heterosexual data), we obtained eight Spearman rank correlations between the each of the eight dependent variables aforementioned (FAST slope differences, four IRAP trial-types, two combined *D*_IRAP_ scores, and the averaged-out *D*_IRAP_ score) and the KSOG. With the exception of the area under the ROC curve (AUC) plots from the *pROC* package, all graphs were generated using the *ggplot2* package (Wickham, 2016). Nearly all observed effect sizes were above the minimum effect sizes we could detect with 80% power.

## Results

**Fig. 2:**
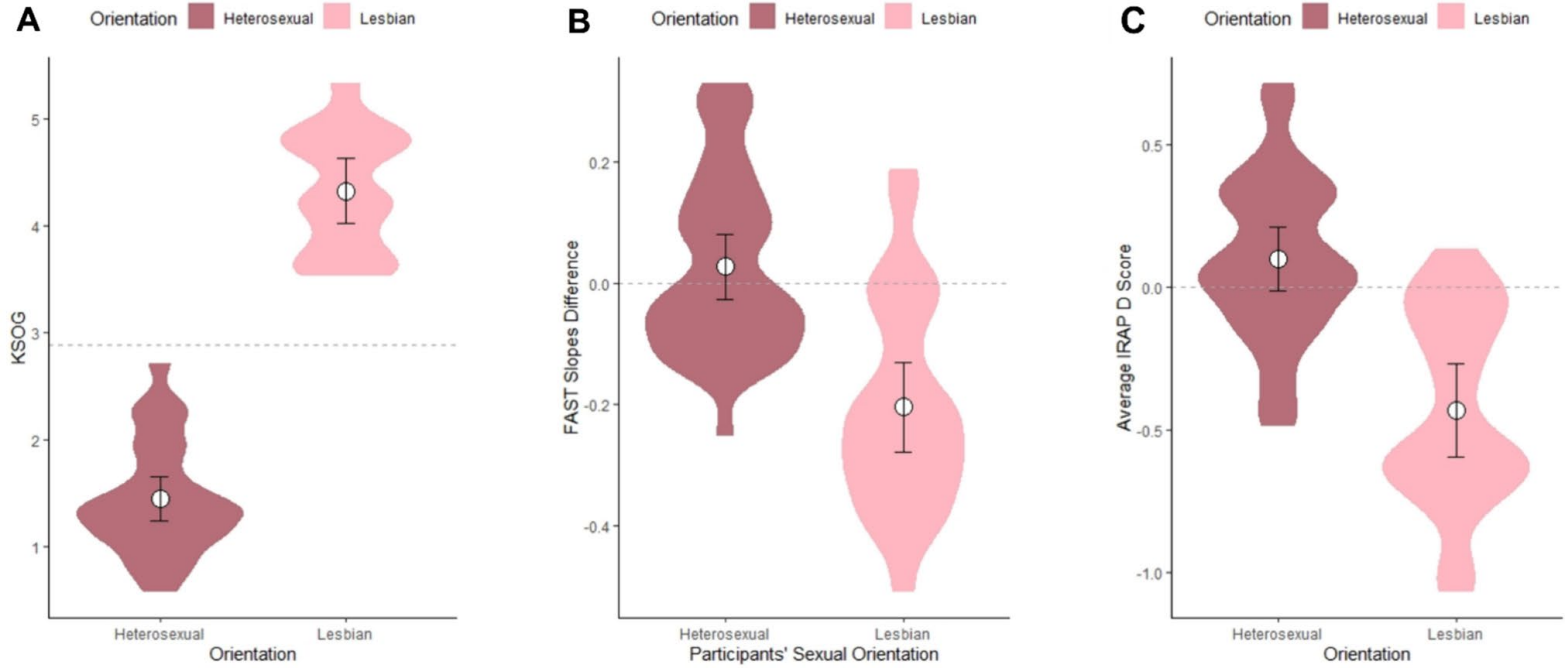
Summary distribution of KSOG, FAST Slopes Differences, and averaged-out *D*_IRAP_ Score, by sexual orientation. *Note*. Panel A: KSOG ordinal data varied from 1 to 7, where 1 indicates strict heterosexuality, 3.5 strict bisexuality, and 7 strict homosexuality. Panel B: Positive FAST slope differences indicate higher learning-curve slopes (i.e., more ease) in “Straight woman” blocks, and negative FAST slope differences indicate higher slopes in “Lesbian woman” blocks. Panel C: Positive average *D*_IRAP_ scores indicate more promptness in relating males to sensual attributes and females to aversive attributes (i.e., “Straight-like responding”), whereas negative average *D*_IRAP_ scores indicate more promptness in relating females to sensual attributes and males to aversive attributes (i.e., “Lesbian-like responding”). No panels share the same *y*-axis scale. The dashed line on panel A represents the weighted mean due to reduced number of lesbian participants without KSOG data (16 versus 28 heterosexuals). The dashed lines on panels B and C represent *y* = 0. White circles represent means. Error bars represent 95% confidence intervals. Wider widths in violin plots represent higher concentration of observations around that *y*-axis level

Of the 58 participants who were exposed to the IRAP, 48 successfully met the inclusion criteria from practice blocks: 28 heterosexual women and 20 lesbian women (i.e., 5 participants in each group failed to meet practice blocks performance criteria). The *D*_IRAP_ scores for these 48 participants, averaged out across the four trial-types, are presented in Fig. 2. Figure 2 also presents the 58 participants’ FAST Slope Difference scores, as well as 54 participants’ KSOG results (four lesbian participants’ KSOG data were lost due to a technical error). Table 1 presents descriptive statistics and pairwise comparisons for the KSOG, FAST slope differences, averaged *D*_IRAP_ score, and *D*_IRAP_ scores per trial-type, contrasted between-subjects based on sexual orientation.

**Table 1:** Between-subjects comparison of descriptive statistics and inferential results for KSOG, FAST slope differences, IRAP averaged *D*_IRAP_ score, and *D*_IRAP_ scores for each IRAP trial-type

Group	Descriptive statistics						Inferential statistics	
	Min	1st quartile	Median	Mean (SD)	3rd quartile	Max	Pairwise comparison	Effect size
*KSOG self-repot ratings*								
Heterosexual	0.570	1.117	1.33	1.44 (0.53)	1.748	2.710	*W* = 0, *p* < .001	*r* = 0 (large),^a^ Cliff’s *δ* = − 1 (large)
Lesbian	3.520	3.822	4.26	4.32 (0.56)	4.785	5.330		
*FAST slope differences*								
Heterosexual	− 0.252	− 0.073	− 0.01	0.02 (0.15)	0.134	0.329	*W* = 682,^b^ *p* < .001	*r* = 0.826 (large),^a,b^ Cliff’s *δ* = 0.653 (large)
Lesbian	− 0.509	− 0.335	− 0.22	− 0.20 (0.17)	− 0.064	0.187		
*D*_*IRAP*_* scores averaged across the four trial-types*								
Heterosexual	− 0.485	− 0.093	0.06	0.10 (0.29)	0.324	0.717	*t*(36.329) = 5.53,^c^ *p* < .001	Cohen’s *d* = 1.66 (large)^c^
Lesbian	− 1.068	− 0.649	− 0.53	− 0.43 (0.35)	− 0.104	0.134		
*IRAP trial-type 1*								
Heterosexual	− 0.795	0.052	0.42	0.40 (0.54)	0.861	1.152	*t*(147) = 2.64,^d^ *p* < .01	Cohen’s *d* = 0.69 (med)
Lesbian	− 0.763	− 0.259	0.06	0.04 (0.47)	0.334	0.921		
*IRAP trial-type 2*								
Heterosexual	− 0.317	− 0.033	0.34	0.28 (0.34)	0.481	0.870	*t*(147) = 5.02,^d^ *p* < .001	Cohen’s *d* = 1.75 (large)
Lesbian	− 1.280	− 0.645	− 0.42	− 0.39 (0.44)	− 0.223	0.549		
*IRAP trial-type 3*								
Heterosexual	− 1.042	− 0.478	− 0.07	− 0.13 (0.50)	0.199	0.987	*t*(147) = 4.76,^d^ *p* < .001	Cohen’s *d* = 1.35 (large)
Lesbian	− 1.468	− 1.142	− 0.81	− 0.78 (0.43)	− 0.477	0.282		
*IRAP trial-type 4*								
Heterosexual	− 0.785	− 0.576	− 0.18	− 0.15 (0.46)	0.214	0.785	*t*(147) = 3.18,^d,e^ *p* < .005	*A* = 0.746 (large),^a,e^ Cliff’s *δ* = 0.493 (large)
Lesbian	− 1.481	− 0.825	− 0.65	− 0.58 (0.45)	− 0.267	0.379		

^a^Our *r* estimator results coincided with results from the Vargha-Delaney’s *A* estimator (from the *effsize* package) classified as large effect sizes

^b^Parametric results (only for comparison with the averaged *D*_IRAP_ score) for FAST slope differences would have been: *t*(46.441) = 5.19, *p* < .001; Cohen’s *d* = 1.41 (large)

^c^Nonparametric results for averaged *D*_IRAP_ score (for comparison with FAST slope differences results): *W* = 491, *p* < .001; Cliff’s *δ* = 0.754 (large); Vargha-Delaney’s *A* = 0.877 (large; effectively equal to *r* = 0.87)

^d^The multiple pairwise between-subjects comparisons were obtained from the linear mixed-effects model fit by restricted maximum likelihood, with Orientation as the between-subjects factor and Trial-type as the within-subjects factor. The Kenward–Roger degrees-of-freedom method for one pair of between-subjects comparison per IRAP trial-type was employed

^e^While the pairwise comparison was in the context of a parametric model, we opted for nonparametric effect size estimators for trial-type 4. This is because, while no *D*_IRAP_ score was technically shown to violate parametric assumptions, heterosexuals’ *D*_IRAP_ scores at trial-type 4 had a marginal Shapiro–Wilk result (*p* = 0.10), and therefore this particular level could be considered to potentially deviate from normality

Lesbian and heterosexual participants’ explicit self-reports in the KSOG did not overlap, and therefore the difference between sexual orientations was highly significant. Because KSOG data are ordinal within a 7-point scale (and due to the data loss), it served only to confirm participants’ self-reported sexual orientation, which will henceforth be treated as a dichotomous variable (except for bivariate rank correlations).

### Function Acquisition Speed Test

Similarly to the explicit measure, FAST slope differences were also shown to be significantly different between sexual orientations (*p* < 0.001, see Table 1). However, heterosexual participants’ FAST Slopes in the “Lesbian woman” and “Straight woman” blocks were not significantly different to one another (Wilcoxon’s *W* = 477, *p* = 0.393, effect size: Cliff’s *δ* =  − 0.124). On the other hand, lesbian participants did perform significantly differently in these two types of blocks (Wilcoxon’s *W* = 456.5, *p* < 0.01, effect size: Cliff’s *δ* = 0.461). Lesbian participants’ mean slope in the “Lesbian woman” block was 0.633, whereas in the “Straight woman” block it was 0.430, thereby indicating that the they performed significantly better in the former block (respond equally for female pictures and positive terms, and for male pictures and negative terms) than the latter (respond equally for male pictures and positive terms, and for female pictures and negative terms).^3^ All in all, while FAST performances were significantly different between groups, and the lesbian women responded “like Lesbians,” the heterosexual women did not show a particular bias for male or female pictures.^4^

### Implicit Relational Assessment Procedure Compared with the Function Acquisition Speed Test

Because the IRAP provides results specific to each possible combination of stimuli (trial-types), the only way to directly compare it to the FAST is by averaging out the *D*_IRAP_ scores across the trial-types. Therefore, before providing results at the trial-type level, we will present IRAP results for the averaged-out *D*_IRAP_ score. As shown in Table 1, the averaged *D*_IRAP_ scores are significantly different (*p* < 0.001) between lesbian and heterosexual participants. While lesbian participants’ averaged *D*_IRAP_ score differed significantly from zero (*t*(19) =  − 5.49, *p* < 0.001, Cohen’s *d* =  − 1.22), heterosexual participants’ did not (*t*(27) = 1.80, *p* = 0.082, Cohen’s *d* = 0.34). Because *D*_IRAP_ scores involve the difference in responding between “Lesbian woman” blocks and “Straight woman” blocks, a difference from zero indicates the same type of result as the comparison of FAST Slopes per block (i.e., both represent the difference between two types of block). Again, while the IRAP differentiated the responding of lesbian and heterosexual participants significantly, and lesbian participants responded “like lesbians” (i.e., performed better in the “Lesbian woman” blocks), heterosexual participants did not significantly differentiate their performance between the two types of blocks.

### Implicit Relational Assessment Procedure-Specific Findings

**Fig. 3:**
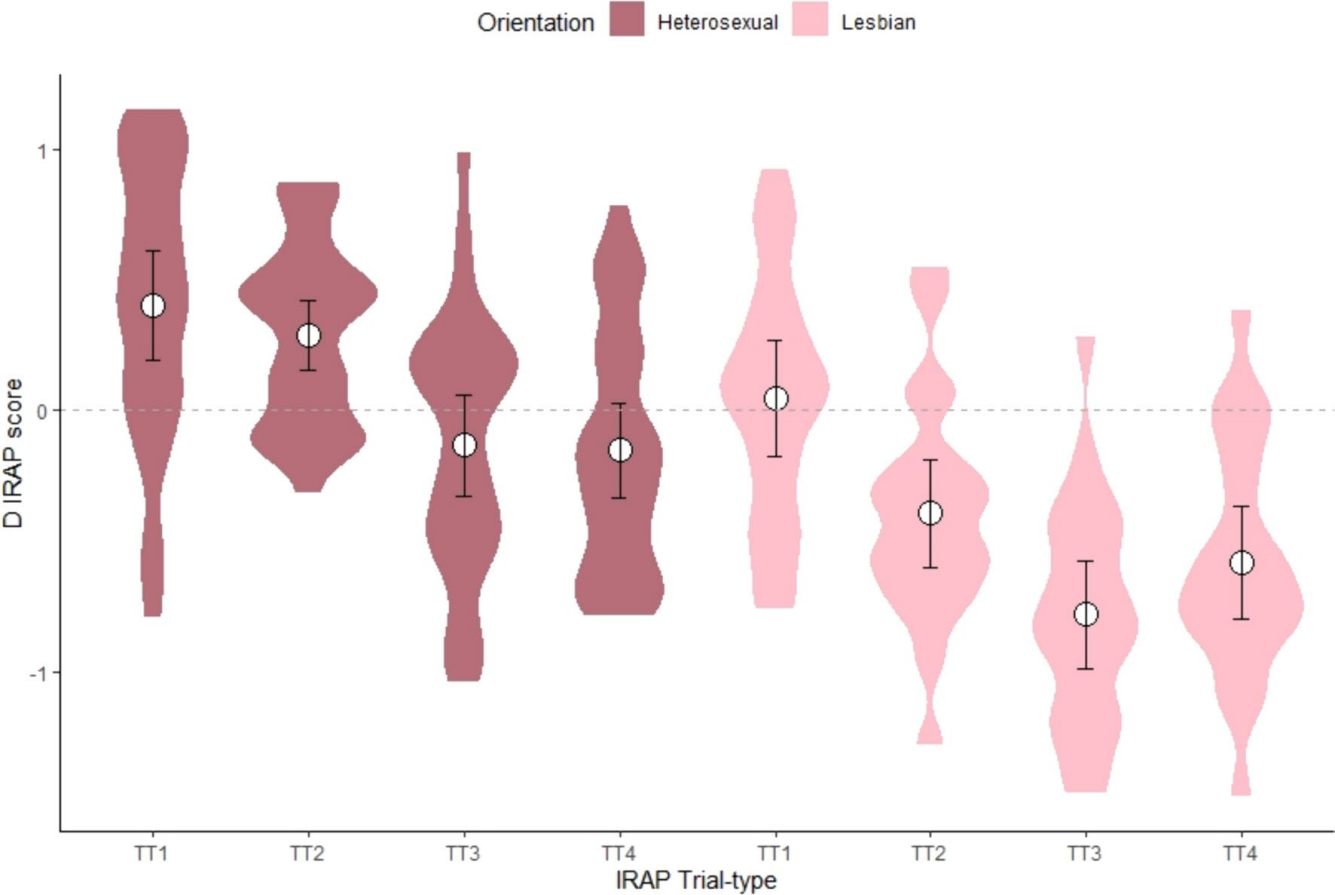
Heterosexual and lesbian participants’ *D*_IRAP_ scores at each of the four trial-types. *Note*. Data from the 48 participants who met IRAP practice criteria. White circles represent means. Error bars represent 95% uncorrected confidence intervals (see Table 3 for the corrected confidence limits). TT = trial-type. Positive values indicate a “heterosexual-like” pattern of responding (i.e., quicker in the “Straight woman” blocks), and negative values indicate a “Lesbian-like” pattern of responding (i.e., quicker in the “Lesbian woman” blocks). Wider widths in violin plots represent higher concentration of observations around that *y*-axis level. In terms of each mean’s difference from zero, in trial-type 1, heterosexual participants had a significant positive *D*_IRAP_ score [*t*(27) = 3.93, *p* < .001] whereas the lesbian participants had a score not significantly different from zero [*t*(19) = 0.41, *p* = .69]; trial type 2 produced a significant positive *D*_IRAP_ score for the heterosexual group [*t*(27) = 4.38, *p* < .001] and a significant negative score for the lesbian group [*t*(19) = 4.0, *p* < .001]; trial type 3 produced a non-significant negative *D*_IRAP_ score for the heterosexual group [*t*(27) = 1.42, *p* = .17] and a significant negative score for the lesbian group [*t*(19) = 7.99, *p* < .001]; trial type 4 produced a non-significant negative *D*_IRAP_ score for the heterosexual group [*t*(27) = 1.72, *p* = .096] and a significant negative score for the lesbian group [*t*(19) = 5.71, *p* < .001]

The IRAP also permits to focus on trial-types: in this case, the specific combinations of male or female pictures with positive or negative attributes (see Fig. 1). The *D*_IRAP_ scores for each of the four trial-types are presented in Fig. 3. Taking *D*_IRAP_ scores as the dependent variable, a linear mixed-effects model was fitted by restricted maximum likelihood, with sexual orientation as the between-subjects fixed factor, Trial-type as the within-subjects fixed factor, and the participants as the random-effects factor. Table 2 shows the type-III ANOVA table for this model. The main effects for sexual orientation and trial-type are both significant, but the interaction is not. This is confirmed when contrasted with a visual inspection of Fig. 3, where the same pattern of trial-type averages (trial-type 1 > trial-type 2 ≫ trial-type 3 < trial-type 4) seems to be replicated across both levels of sexual orientation. Between-subjects pairwise comparisons are significant at the *α* = 0.01 level for all trial-types (see Table 1), with all heterosexuals’ *D*_IRAP_ scores higher than lesbians’ *D*_IRAP_ scores at each trial-type. For both groups, negative *D*_IRAP_ scores indicate “Lesbian-like” responding, and positive *D*_IRAP_ scores indicate “Straight-like” responding. Therefore, trial-type results interpretation needs to take this into account. As expected (see Fig. 1), the “dominant” positive-mean trial-type for heterosexuals was trial-type 1, whereas the “dominant” negative-mean trial-type for lesbians was trial-type 3.

**Table 2:** Type III analysis of variance table with Satterthwaite’s method

	Sum Sq	Mean Sq	d.f. (num.)	d.f. (den.)	*F*	*p*
Trial-type	13.553	4.518	3	138	29.662	7 × 10^−15^
Sexual orientation	4.948	4.948	1	46	32.486	8 × 10^−07^
Interaction	0.882	0.294	3	138	1.931	0.128

**Fig. 4:**
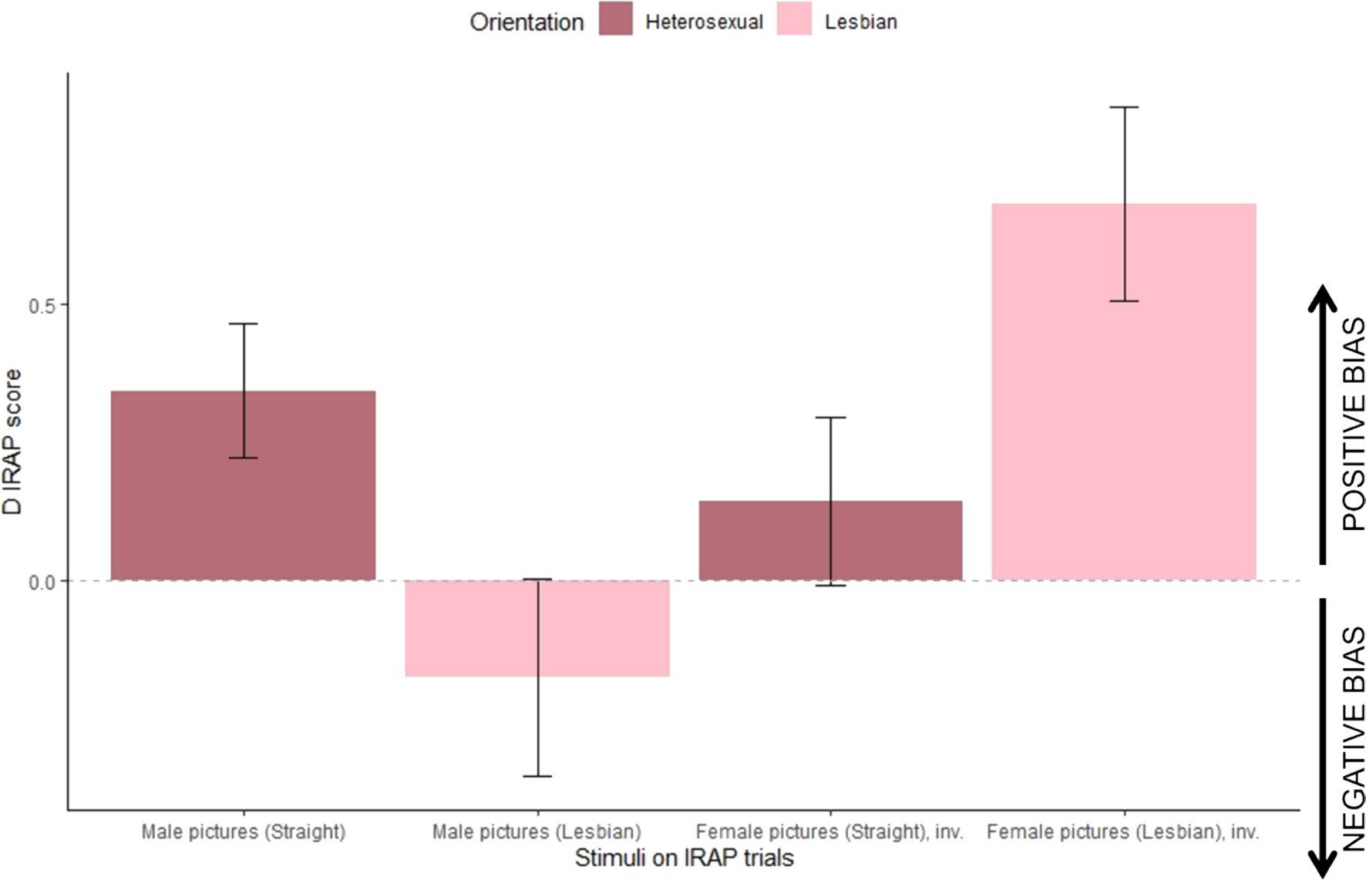
Heterosexual and Lesbian participants’ *D*_IRAP_ scores at each of the four trial-types. *Note*. Bar heights represent means. Originally, positive *D*_IRAP_ scores represent a “heterosexual-like” pattern of responding, and negative *D*_IRAP_ scores a “Lesbian-like” pattern of responding. However, in this figure we inverted *D*_IRAP_ scores in trial-types 3 and 4 (female pictures) by multiplying them to − 1, thereby rendering all positive scores positive biases (i.e., quicker to relate the pictures with positive terms as “true” and with negative terms as “false”), and all negative scores negative biases (i.e., quicker to relate the pictures with negative terms as “true” and with positive terms as “false”). The first two bars collapsed trial-types 1 and 2 (male pictures) and the last two bars collapsed trial-types 3 and 4 (female pictures). Error whiskers represent uncorrected 95% confidence intervals

Table 3 presents within-subjects pairwise *D*_IRAP_ scores comparisons across the six possible two-by-two combinations of trial-types for each sexual orientation.^5^ Within the heterosexual group, the pairwise contrasts that are not significantly different are trial-type 1 with trial-type 2 and trial-type 3 with trial-type 4. All other contrasts are significant at the *α* = 0.001 level. Within the lesbian group, the pairwise comparisons that were not significant were between 2 and 4, and between 3 and 4. Trial-type 1 was significantly different from trial-types 3 and 4 at the *α* = 0.001 level, but significantly different from trial-type 2 at the *α* = 0.01 level. Trial-types 2 and 3 were significantly different at the *α* = 0.05 level. Importantly, the lesbian dominant trial-type was as far from zero as the other trial-type involving female pictures, thereby indicating a type of “pro-women” bias that was *not* mirrored as an “anti-women” bias in heterosexual participants (*D*_IRAP_ scores in heterosexuals’ trial-types 3 and 4 are not different from zero). Based on this, it is worth combining the two trial-types that involve male pictures (i.e., 1 and 2) and the two that involve female pictures (i.e., 3 and 4), to study the broader “pro-male” or “pro-female” biases between the two groups. To map onto Timmins et al.’s (2016) Fig. 2, we have inverted the trial-types whose negative scores indicated a more “pro-female” bias (3 and 4 in our case), and presented means as positive or negative biases, as shown in Fig. 4.^6^ Heterosexuals seem to display a “pro-male” bias, and lesbians a (higher) “pro-female” bias. Lesbian participants seem not to show any particular bias to male pictures, nor heterosexual participants to female pictures. However, on average (albeit weak), lesbian participants indicated an “anti-male” bias, whereas heterosexuals did not indicate an “anti-female” bias.

**Table 3:** Within-subjects pairwise comparisons of *D*_IRAP_ scores per trial-type

	95% CI (Estimated mean)	Contrasts			
		Trial-type 1	Trial-type 2	Trial-type 3	Trial-type 4
*Heterosexual group*					
Trial-type 1	[0.159, 0.645]	–			
Trial-type 2	[0.044, 0.530]	*t*(138) = 1.10, *p* = .690	–		
Trial-type 3	[− 0.379, 0.108]	*t*(138) = 5.15, *p* < .001	*t*(138) = 4.04, *p* < .001	–	
Trial-type 4	[− 0.396, 0.090]	*t*(138) = 5.31, *p* < .001	*t*(138) = 4.21, *p* < .001	*t*(138) = 0.16, *p* = .998	–
*Lesbian group*					
Trial-type 1	[− 0.244, 0.331]	–			
Trial-type 2	[− 0.682, − 0.107]	*t*(138) = 3.54, *p* = .003	–		
Trial-type 3	[− 1.070, − 0.495]	*t*(138) = 6.68, *p* .001	*t*(138) = 3.14, *p* = .011	–	
Trial-type 4	[− 0.872, − 0.297]	*t*(138) = 5.09, *p* < .001	*t*(138) = 1.54, *p* = .415	*t*(138) = − 1.59, *p* = .383	–

Estimated means’ 95% confidence intervals employed Kenward–Roger degrees-of-freedom estimator (estimate = 147) with Bonferroni adjustment for eight estimates. If zero is contained within the interval, the participants’ pattern of responses in the “Lesbian woman” and “Straight woman” blocks were not significantly different. Pairwise contrast inferences employed Kenward–Roger degrees-of-freedom estimator (estimate = 138) with Tukey *p*-value adjustment method for comparing a family of four estimates

### Area Under ROC Curves

**Fig. 5:**
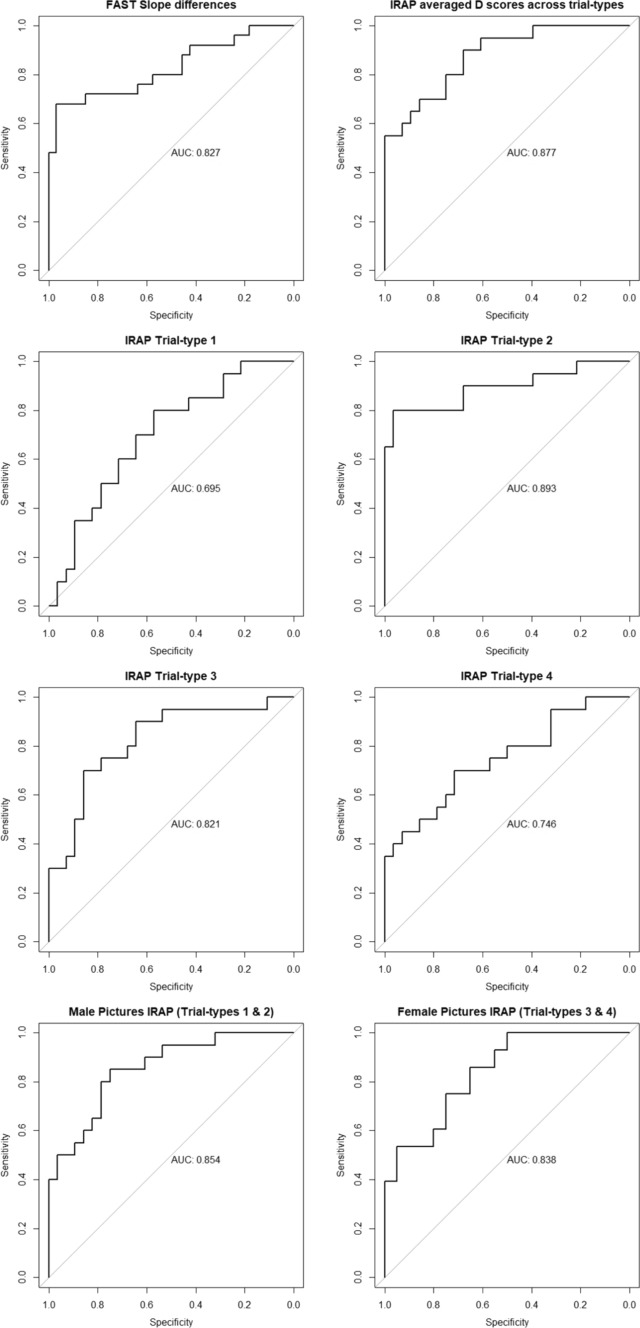
Area under the ROC curve for identifying lesbian and heterosexual women based on FAST slope differences and the IRAP (averaged-out *D*_IRAP_ scores and *D*_IRAP_ scores per trial-type and per picture gender). *Note*. For all ROC curves, the direction was controls > cases, where cases were lesbian participants and controls were heterosexual participants. However, for the female pictures IRAP (combined trial-types 3 and 4), because the variable was inverted, we set the heterosexual level of the sexual orientation factor to be the case, effectively maintaining it as control and the lesbian level as case. The *y*-axis represented sensitivity, or the true-positive rate, and the *x*-axis represented specificity, or the true-negative rate, which ranges from 1 at the origin to 0 at the right-tail. This *x*-axis is therefore the inverse of that depicted by Timmins et al. (2016), in which the false positive rate was plotted along the *x*-axis from 0 sat the origin up until 1 at the right-tail

Figure 5 presents the area under the receiver operating characteristic (ROC) curve (AUC) obtained for the sensitivity in detecting lesbian participants against specificity. One curve is presented for each one of the dependent variables hitherto described. In comparing FAST slope differences and averaged-out *D*_IRAP_ scores, the sensitivity–specificity trade-off of the IRAP (AUC = 0.877) outperformed slightly that of the FAST (AUC = 0.827), but not significantly (DeLong’s *D*_(103.24)_ = 0.654, *p* = 0.515). The IRAP trial-type that best discriminated between sexual orientations was trial-type 2 (AUC = 0.893), and the weakest was trial-type 1 (AUC = 0.695). In the IRAP, male pictures (AUC = 0.854) narrowly outperformed female pictures (AUC = 0.838) in identifying Lesbian among heterosexual women, but the difference was not significant (DeLong’s *D*_(93.74)_ = 0.203, *p* = 0.840).

### Correlating the Klein Sexual Orientation Grid with the Function Acquisition Speed Test and Implicit Relational Assessment Procedure Measures

For all analyses heretofore described, sexual orientation was considered a dichotomous factor due to the nature of the KSOG scale. Nevertheless, we henceforth describe putative nonparametric correlations between the dependent variables and the KSOG (which is non-normally and seemingly bimodally distributed). Spearman’s rank correlation coefficient *ρ* was significantly different from zero at the *α* = 0.02 level between the KSOG and all other variables, namely: FAST slope differences (*ρ* =  − 0.591, *p* < 0.001), averaged-out *D*_IRAP_ score (*ρ* =  − 0.687, *p* < 0.001), IRAP trial-type 1 (*ρ* =  − 0.377, *p* = 0.012), IRAP trial-type 2 (*ρ* =  − 0.582, *p* < 0.001), IRAP trial-type 3 (*ρ* =  − 0.473, *p* < 0.005), IRAP trial-type 4 (*ρ* =  − 0.629, *p* < 0.001), IRAP male pictures (*ρ* =  − 0.608, *p* < 0.001), and IRAP female pictures^7^ (*ρ* =  − 0.614, *p* < 0.001). Albeit significant, the strength of the coefficients ranged noticeably from weak (trial-type 1) to relatively strong (averaged-out IRAP), but none were extremely strong. All correlation coefficients are negative, since higher values in the KSOG indicate a lesbian orientation, whereas negative values in both FAST slope differences and *D*_IRAP_ scores indicate better fluency in “Lesbian woman” blocks (see Fig. 2).

## Discussion

In the present study, both the IRAP and the FAST showed relative success in differentiating women’s sexual orientation when employing a sample of lesbian and heterosexual participants. However, participants did not distinguish their performance in an orthogonal fashion when responding to male and female pictures and negative and positive attributes. In both implicit measures, while the pattern of responding was significantly different between the two orientations, heterosexual women did not show a significant difference in the two different ways they were requested to perform (namely, “heterosexual-like” or “lesbian-like”), whereas lesbian participants did perform better when required to demonstrate preference for women and repulsiveness to men than when the opposite pattern was required.

In the IRAP, significant differences were observed between heterosexual and lesbian women’s responses. The study distinguished trial-types between “Lesbian woman” and “Straight woman” blocks, with consistent blocks aligning with participants’ sexual orientations. For heterosexual women, trial-type 1 showed maximum coherence, whereas for lesbian women, it was trial-type 3. Trial-types 1 and 2 for heterosexuals and 3 and 4 for lesbians featured positive images. Positive IRAP scores indicated a “Straight-like” bias, while negative scores indicated a “Lesbian-like” bias. Heterosexual participants showed significant differences in responses between genders, with positive effects for males. Interestingly, heterosexual women showed a relative positive bias in responding to female nudes, with marginally negative *D*_IRAP_ scores in trial-types 3 and 4, coherent with a “Lesbian-like” response pattern, though this did not reach statistical significance. Lesbians displayed significant “Lesbian-like” effects in all but trial-type 1, particularly in trial-types 2, 3, and 4. The trial-type 2 most effectively differentiated between sexual orientations. Heterosexuals were quicker to deny male repulsiveness, while lesbians accepted it. Overall, the IRAP was effective in distinguishing between the two groups by averaging responses across trial-types.

The FAST is also effective in differentiating the sexual orientations—slightly less so than the IRAP, but not significantly. Unlike the IRAP, the FAST does not permit to explore the specific combinations of stimuli. By comparing participants’ ease in acquiring the same behavioral function between category stimuli and positive evaluative stimuli to that in acquiring the same function between the same category and negative evaluations, it simply allows to estimate the strength of previous relations between category and evaluation, but not the specific nature of this relation. In the IRAP, according to the DAARRE model, this is the *C*_*rel*_ property of each trial-type. One advantage of the FAST is that it requires much less from the participants in terms of time and effort. Taking this trade-off into account, and because it is not significantly worse than the averaged-out IRAP, the FAST can be used as a “quick-and-dirty” way to differentiate sexual orientations, if there are no specific hypotheses about the nature of the positivity or negativity in approaching or avoiding certain genders to be tested. However, with the DAARRE model, these particular relations can be explored in light of the influence of the psychological impacts of each stimuli presented per trial—but only in the IRAP.

For example, it seems like the female figure does not exactly bear such a − *C*_*func*_ as originally hypothesized for the heterosexual participants, and perhaps the + *C*_*func*_ of positive attributes played an even bigger role for lesbian participants than expected. The psychological functions represented as *C*_*func*_ can be of a range of different domains, such as attentional or emotional. It may be, therefore, that the female figures for heterosexual participants stand out attentionally (Snowden et al., 2016) or that they have approachability qualities. The orientational/attentional conjecture seems more reliable in face of the studies with male participants. While the gender-non-specificity was hereby replicated with heterosexual women in both the IRAP and the FAST, only in the IRAP can we compare gay men’s disconformity of female attractiveness (Rönspies et al., 2015) with lesbians’ conformity of male repulsion.

Coherent with gay men’s previously observed lack of IRAP effects to the sensuality of women, heterosexual women’ non-orthogonal IRAP performances hereby observed could be attributed to the frequent depiction of women as sexual objects in media. This portrayal, often emphasizing women’s greater sexual appeal in advertisements where female models are more attractive and younger than male models (Lin, 1998), might reduce the heterosexual women’s implicit bias against female images. We are speculating here that such consistent exposure to the female figure as sexualized beings potentially influences women’s automatic reactions to female stimuli, thereby mitigating heterosexual women’s opposition to women as sexually attractive and corroborating lesbian women’s automatic biases in coherence with their self-reported sexual orientation. In line with the DAARRE model and previous implicit evidence with men, the eroticization of the feminine may expand not only men’s but also women’s relational networks relating gender figures to sexual response, enhancing lesbian women and heterosexual men’s relational responses of attraction to females, and diminishing heterosexual women and gay men’s aversion to the feminine figure.

One corollary of this female objectification conjecture is the putative development of relational networks connecting sexuality and femininity through observation by heterosexual women and gay men, even in the absence of direct sexual experiences with women. In support of this assumption, Dawson et al. (2012) reported a significant incidence of same-gender fantasy among heterosexual women. However, the connection between such fantasies or exposure to sexualized women in the media and non-specific sexual responses in heterosexual women remains largely unexplored and speculative.

One reason why heterosexual women may lack gender-specific biases is that they respond genitally to sexual activity itself more so than to the category of the actors, and respond equally to solitary male and female nudes depicted exercising or masturbating, whereas lesbian women differentiate their genital responses toward solitary females over males (Chivers et al., 2007). This pattern of response to activity over gender was confirmed not to be dependent on menstrual cycle phase in heterosexual women (Bossio et al., 2014). Physiological methods of measurement of genital arousal evidently differ between males and females, which is one possible explanation for differences between sexes in showing gender-related sexual responses. However, in the present study, both implicit measures involved solely pressing keys on the keyboard, which is assumed to involve the exact same process across sexes, and the same pattern was relatively confirmed. This pattern of heterosexual women gender-non-specificity has been confirmed with other non-genital measures, such as pupil dilatation (Holmes et al., 2021; Rieger & Savin-Williams, 2012) and other implicit measures (e.g., Snowden et al., 2016), such as the Implicit Associations Test (Snowden & Gray, 2013; Snowden et al., 2024). It seems like the behavioral tasks’ advantage of wielding procedural coherence across sexes does not make them any less valid than genital measures, and is replicated in the “quicker” FAST and the “more specific” IRAP. While the FAST takes much less time and demands less from participants, the IRAP is nevertheless the only instrument with the advantage of enabling researchers to look deeper into all relational combinations across two pairs of stimuli (e.g., male, female, attractive, unattractive).

The present study replicated relatively not only the category-non-specificity of heterosexual women but also the category-specificity of lesbians (Rullo et al., 2010). In the context of sexual orientation, the IRAP had only been used with male participants. In contrasting the present results with those of Timmins et al. (2016), we have observed a category-non-specific bias in heterosexual women, whereas Timmins et al. observed category-specificity in heterosexual men, and while both gay women and men showed a strong bias in favor of their loved gender, lesbian women did seem to show a very slight “anti-men” bias, whereas gay men did not show any bias for women. Rönspies et al. (2015) showed this to be the case because, more specifically, gay men were equally likely to confirm and disconfirm sexual unattractiveness to women, but showed a strong effect in disconfirming that women were attractive. In the present study, we observed the reversed pattern in lesbian participants: they were equally likely to confirm and disconfirm attractiveness to men, but showed a strong effect in confirming that men were unattractive.

One important procedural difference between the present study and that of Timmins et al. (2016) is that the response options were presented in fixed positions in our IRAP, whereas they randomly swapped positions in the former study. One difference to Rönspies et al.’s (2015) study is that by not randomizing block order sequence, they effectively started all heterosexual men with their history-coherent block and all gay men with their history-incoherent block, whereas both Timmins et al. and our study counterbalanced block orders across participants. Across the three studies, a fixed sequence of procedures were applied to all participants. One question that remains is whether counterbalancing the order of distinct implicit measures (apart from the IRAP, the FAST in our case and VT in Rönspies et al.) would produce any difference in implicit biases. Another issue that may have been relevant with female participants is to check whether they have been using oral hormonal contraception at the time of data collection, which may affect attractiveness gender-specificity (Little et al., 2013; Rupp & Wallen, 2007).

All in all, the present study employed the IRAP and the FAST with heterosexual and lesbian women for the first time. In doing so, we replicated the typical effects observed in the literature. Namely, in both implicit measures, the heterosexual participants did not perform significantly differently in the “Straight woman” and “Lesbian woman” blocks, while the lesbian participants did perform significantly better in the latter than the former. While both measures allow for a comparison between two types of blocks, only the IRAP permits to explore the nature of specific relations between a gender figure and approaching/avoiding attributes—that is, specific relations in a relational network. Nevertheless, both implicit measures were relatively successful in differentiating the two sexual orientations as a group. They did not, however, exactly map onto self-reported sexual orientation, which is in line with previous studies using genital measures (Chivers et al., 2015; Suschinsky et al., 2017). One could argue that perhaps genital measures are not all there is to sexual orientation, but the present study goes one level further in employing implicit behavioral measures that confirm such patterns observed in genital responses.

## Data Availability

All data and analyses code used in this research are available on OSF at https://osf.io/4vybr/ in a public project.

## Footnotes

1 The abbreviation “V” usually stands for “Verdadeiro” in the Brazilian context, which translates to “True” in English; same for “F” (“Falso”).

2 These estimates were effectively equivalent to the Vargha-Delaney *A* estimates in our sample.

3 For the heterosexual participants, Slope in the “Lesbian woman” block was 0.622, and in the “Straight woman” block it was 0.659 (i.e., nearly no difference).

4 If we could adjust a parametric linear model to FAST slope differences, we would reach the same conclusion by observing that the estimated marginal mean with 56 degrees-of-freedom for heterosexual participants falls within a 95% Confidence Interval between − 0.039 and 0.092 (i.e., including zero), and for lesbian participants between − 0.279 and − 0.128, with Bonferroni confidence level adjustment for two estimates.

5 Along with 95% confidence interval estimates for the mean of each trial-type per sexual orientation.

6 There were some differences between our Fig. 4 and Timmins et al.’s Fig. 2. The error bars in our figure represent 95% CI rather than SEM. Because we are dealing with gay and straight women rather than men, we plotted the male pictures first and the female pictures last. It was the female pictures that were inverted to represent a positive bias rather than a heterosexual-like responding, instead of the male pictures in the case of Timmins et al. (2016).

7 Because we used the inverted data, we actually observed the positive *ρ* = 0.614, but had the *D*_IRAP_ scores not been multiplied by − 1, the correlation coefficient would have been negative like all others.

## Code Availability

The data-analysis code can be found on OSF in the same project as the data.
